# Associations of Diet with Health Outcomes in the UK Biobank: A Systematic Review

**DOI:** 10.3390/nu16040523

**Published:** 2024-02-13

**Authors:** Hana F. Navratilova, Susan Lanham-New, Anthony D. Whetton, Nophar Geifman

**Affiliations:** 1 School of Biosciences, Faculty of Health and Medical Sciences, University of Surrey, Guildford GU2 7XH, UK; h.navratilova@surrey.ac.uk (H.F.N.); s.lanham-new@surrey.ac.uk (S.L.-N.); a.whetton@surrey.ac.uk (A.D.W.); 2 School of Health Sciences, Faculty of Health and Medical Sciences, University of Surrey, Guildford GU2 7YH, UK; 3 Department of Community Nutrition, Faculty of Human Ecology, IPB University, Bogor 16680, Indonesia; 4 Veterinary Health Innovation Engine, School of Veterinary Medicine, University of Surrey, Guildford GU2 7XH, UK

**Keywords:** cardiovascular diseases, cancer, diabetes mellitus, dietary assessment, food frequency questionnaire, food preference questionnaire, online 24 h dietary assessment, middle aged, UK Biobank

## Abstract

The UK Biobank is a cohort study that collects data on diet, lifestyle, biomarkers, and health to examine diet–disease associations. Based on the UK Biobank, we reviewed 36 studies on diet and three health conditions: type 2 diabetes (T2DM), cardiovascular disease (CVD), and cancer. Most studies used one-time dietary data instead of repeated 24 h recalls, which may lead to measurement errors and bias in estimating diet–disease associations. We also found that most studies focused on single food groups or macronutrients, while few studies adopted a dietary pattern approach. Several studies consistently showed that eating more red and processed meat led to a higher risk of lung and colorectal cancer. The results suggest that high adherence to “healthy” dietary patterns (consuming various food types, with at least three servings/day of whole grain, fruits, and vegetables, and meat and processed meat less than twice a week) slightly lowers the risk of T2DM, CVD, and colorectal cancer. Future research should use multi-omics data and machine learning models to account for the complexity and interactions of dietary components and their effects on disease risk.

## 1. Introduction

Diet is a modifiable risk factor that has been shown to have a significant impact on the prevention and management of chronic diseases. Information on dietary patterns has been observed to help predict cardiovascular disease and find a dietary association with cancers [1,2]. Understanding how diet influences disease risk requires a holistic and integrative approach that considers other factors, such as genetic, environmental, psychological, and behavioural parameters. Large-scale epidemiological studies such as the UK Biobank, where big data related to all those factors are readily available for analysis, are expected to provide better insight into the diet–disease relationship [3].

However, diet as an exposure variable is challenging to measure, as consumption of food happens in varying combinations and proportions, rather than as individual nutrients or food items. In addition, nutrients and foods can interact in synergistic and antagonistic directions [4]. Given this complexity, diet as a predictor of disease has, as is appropriate, been explored in different ways: individual nutrients, food indices, food groups, or dietary patterns. A major problem with categorising diet variability is the limited comparability and generalisability of findings across studies [5].

Given the undoubted benefits of the UK Biobank for understanding diet–disease associations (due in part to the huge cohort size), a quantitative and qualitative evaluation of existing studies is needed to gain a synoptic overview of progress using these data. We conducted a systematic review to identify the knowledge gap regarding the associations between diet and non-communicable disease (NCDs) incidence in UK Biobank participants. The objectives of this paper are to summarise the main findings from the published studies that analysed the UK Biobank dietary data; create summary statistics of the effect of diet/food; and identify the gaps in the current literature to suggest directions for future research.

## 2. Methods

### 2.1. Study Design and Setting

The UK Biobank is a large-scale epidemiological prospective cohort with an overarching objective to serve as a resource for research on the genetic, environmental, and lifestyle factors that influence a wide range of diseases [3]. The UK Biobank recruited 502,000 participants aged 40 to 69. This age group was chosen to enable the study of a population that is susceptible to various chronic conditions (such as cancer, CVD, and diabetes) over the ensuing period of their lives. Data on participants’ biomedical attributes, genome, wellbeing, and diseases were gathered from 2006 to 2010 at 22 recruiting centres across the United Kingdom. Fry et al. found that women, older people, and those living in less deprived areas had higher participation rates. UK Biobank participants also had fewer health problems, lower obesity rates, and less daily alcohol consumption than the general population [6]. The UK Biobank had the same percentage of ethnic diversity that reflects the national proportion [7]. 

### 2.2. Search Strategy and Data Source

PubMed and Web of Science were searched for studies published between 2018 and 2022 using the following terms: UK Biobank, diet*. Articles that met the following eligibility criteria were included: conducted in the UK Biobank using Food Frequency Questionnaire (FFQ), 24 h Oxford WebQ, and/or Food Preference Questionnaire (FPQ); assessed individual foods (e.g., oranges or red meat), complex dietary patterns (e.g., Mediterranean diet or plant-based diet), a priori-defined diet/health indices (e.g., healthful plant-based diet index), and/or specific nutrients (e.g., trans fats) as predictor variables; and examined the relation of food to NCDs in which the environmental factor is known as a risk factor (e.g., cancer, diabetes, cardiovascular disease, hypertension). We excluded studies that focused on food intake for deficiency diseases; investigated the effect of food on NCDs related to cognitive or brain function (such as dementia or Alzheimer’s); or were the only one of their kind for examining a disease, so we could not compare their findings with other relevant studies. Studies combining the UK Biobank with other cohorts were included in this review. 

### 2.3. Data Extraction and Quality Assessment

Data on the effect size of selected studies were compiled in Excel 2016 (Microsoft, Redmond, WA, USA). The Newcastle–Ottawa scale was used to assess the quality of studies included in the summary statistic of effect estimates [8], which assigns a maximum of 9 points to each study. We assigned a quality score to each study based on the following criteria: 0–3 points for low quality, 4–6 points for moderate quality, and 7–9 points for high quality.

### 2.4. Data Analysis and Presentation of Results

The summary of effect estimates combines results from several studies that reported the hazard ratios (HRs) and 95% confidence intervals (CIs) of maximally adjusted models for similar diet exposures. For studies that categorised healthy dietary pattern adherence using dietary indices, we used the risk estimate that compared the highest to the lowest adherence category. When studies used specific dietary patterns (e.g., “healthy” vs. “unhealthy”), we focused on the pattern defined as “healthy” and compared its risk estimate to the “unhealthy” dietary pattern. For studies reporting varying intake proportions, we used the risk estimate that compared the highest to the lowest/no intake. For studies that reported the effect as an odds ratio (OR), the value was converted to an HR following an optimal calculation as described elsewhere [9]. Bubble plots were created to visually display the distribution of effects. In each bubble, the y-axis represented the effect size, while the x-axis represented the dietary focus. The colours of the bubbles indicated the level of significance (green for statistically significant results (*p* < 0.05), red for statistically nonsignificant results (*p* ≥ 0.05), black for cases where no *p*-value was reported). Descriptive statistics (median and interquartile range) were used to summarise the effect across these studies. Additionally, box-and-whisker plots were employed to assess whether the distribution of effects varied by disease type. All analyses were performed using R version 4.2.1 (R Foundation for Statistical Computing, Vienna, Austria) and RStudio version 2023.06.1-524 (RStudio, Boston, MA, USA).

## 3. Results

### 3.1. Dietary Studies Conducted on the UK Biobank

**Figure 1:**
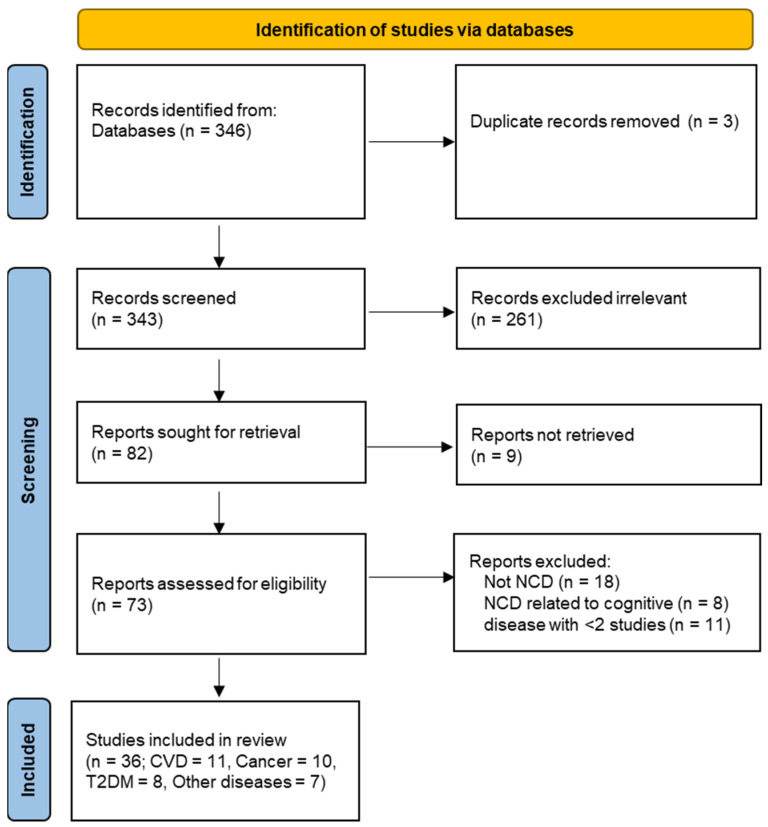
Selection of studies for the systematic review.

Initial searches found 346 potential studies for inclusion. Following selection, we reviewed the findings from 36 relevant studies meeting the criteria set out in the Methods, consisting of eleven studies of CVD, ten studies of cancer, eight studies of T2DM, and seven studies of other NCDs (Figure 1). 

The characteristics of the studies meeting our criteria are presented in Table 1, with the number of participants ranging from 5000 to 400,000 depending on the number of cases of incidence. All studies used an observatory design, including Mendelian randomisation. The quality assessment using the Newcastle–Ottawa scale revealed that the studies included in the summary statistic of effect estimates are categorised as high-quality with only one medium-quality study (score = 6).

### 3.2. Dietary Assessments in the UK Biobank

The UK Biobank integrated the Food Frequency Questionnaire (FFQ) into a baseline touch-screen questionnaire to record participants’ usual intake over a year. There were 29 items about diet in the touch-screen questionnaire, which mainly focused on the frequency of consumption of specific food groups. Dietary data were collected using the FFQ during the initial assessment visit (2006–2010). Several studies have utilised UK Biobank dietary data from the FFQ to find an association of disease risk not only with individual food types [19,21,24,26,28,39,41,42,44], but also with dietary patterns [13,29,31] and dietary indices [16,17,30,35,37,40]. 

The second dietary assessment is the Oxford WebQ, a web-based 24 h dietary recall instrument, developed for UK Biobank studies, which has been validated [46,47] and used for multiday dietary intake assessment [48]. The 24 h dietary recall data were collected during baseline volunteer interviews (April 2009 to September 2009) and collection was repeated at four different time points to assess seasonal variations. It is hard to derive quantitative nutrient intake data on a per-day basis from the FFQ, even when using a semi-quantitative FFQ where the portion size for each consumption is queried. Thus, several studies have combined FFQ with 24 h dietary recall to adjust for individual energy and nutrient intake [22,23,25,27,38]. Measurement bias could arise from the lack of repeated 24 h recalls. For a study that addresses a particular nutrient, especially micronutrients and supplements, it is important to use data from multiple days because a discrepancy between actual intake and reported intake is likely to occur as a result of supplements, or micronutrient-rich food sources, that are usually consumed just a few times per week and not every day [49]. The minimum number of repeated days required to adequately estimate nutrient intake is 4–8 days and varies depending on gender, BMI, and age group [50,51,52]. Carter et al. [53] suggested that two repeated measurements are acceptable to give a better strength of diet–disease associations. However, only a few have used a minimum of two repeated datasets [10,18,23,32,33,45]. 

It may be more convenient and precise to recall food preferences than to recall consumption, making it preferable to evaluate dietary behaviours. The UK Biobank developed the third dietary assessment, the Food Preference Questionnaire (FPQ), with food items not only typical for the UK population but also commonly found worldwide. The questionnaire consists of 150 items, including food items that reflect foodstuff preferences (fruit, vegetables, meat, etc.), sensory preferences (bitter, sweet, etc.), and factors associated with healthy behaviour (exercise, smoking, television viewing, etc.). The preference was measured using a 9-point hedonic scale. The UK Biobank participants were invited to complete the FPQ in 2019. None of the studies included in this review used the FPQ for assessing diet–disease relationships. This leaves an opportunity for researchers to utilise this tool in future investigations.

In contrast to CVD, which largely used data from the 24 h dietary recall, researchers with cancer as their main health outcome mainly used FFQ to find an association. Previously, Kristal et al. [54] pointed out the low level of association between diet and cancer made by FFQ. This is evident from the inconsistent findings of the UK Biobank study, which found different associations between food groups and the same type of cancer. For example, meat intake and lung cancer statistically correlated in some studies [21,22] but not others [23]. 

Several other non-communicable diseases have been studied, including gout [39,40], inflammatory bowel diseases [41,42,43], and rheumatoid arthritis [44,45]. In most of these studies, to find a correlation between different food groups and diseases, FFQ is used for dietary assessment. With animal-protein-based food becoming a common focus, the association with the diseases observed was often mild. Therefore, it remains unclear which diet/food group has the most beneficial effect on health.

### 3.3. Summary of the Effect Estimates of Diet on Health Outcomes in the UK Biobank

Cardiovascular disease (CVD), cancer, and type 2 diabetes mellitus (T2DM) are among the leading causes of morbidity and mortality worldwide. Many researchers using UK Biobank data have studied how diet can help prevent these conditions. This section briefly reviews the studies on diet and these diseases, and then summarises the most consistent and significant findings on specific diet/food types.

#### 3.3.1. Cardiovascular Disease (CVD)

**Figure 2:**
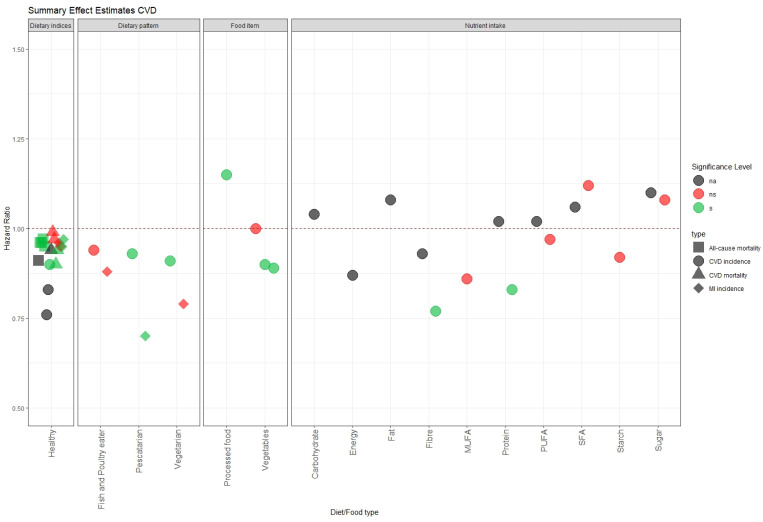
Summary of hazard ratio for the association between food/diet and CVD risk. The points represent different dietary patterns or food items or nutrients and types of CVD risk. The shape shows what kind of CVD outcome was studied (square for all-cause mortality; circle for CVD incidence; triangle for CVD mortality; diamond for myocardial infarction (MI) incidence). An HR of 1 (horizontal red dashed line) means no association. The colour of the points shows the significance level (grey for na = *p*-value not reported; red for ns = *p*-value ≥ 0.05; green for s = *p* value < 0.05).

Dietary indices are often the parameter used to measure diet and CVD incidence in UK Biobank studies (Figure 2). Dietary pattern observations, as opposed to data on a single food group, tend to offer more conclusive findings across the studies, in which a healthy diet is found to be associated with a decrease in CVD incidence and mortality [10,11,12,13,15,16,17,18]. There are several definitions of a healthy diet used in these studies. These studies emphasise a high consumption of plant-based foods, a moderate intake of animal-based foods, and a limited intake of refined grains, processed meats, sugar-sweetened beverages, and foods high in sodium, saturated fat, and added sugars as the most used characteristics [12,16,17,18]. Others focused on a plant-based diet [11,15]. 

When the basis of association is on a single food group or macronutrient, the outcome can be different for the same type of food. One study found an association between processed food and CVD incidence but not CVD mortality [14]. Feng et al. [19] found a benefit of consuming raw vegetables to decrease CVD incidence risk but no benefit of cooked vegetables. Although two studies agree on the effect of a high intake of fibre, sugar, and saturated fatty acids (SFAs), their results are not conclusive [10,20]. Ho et al. [20] reported that macronutrients have non-linear associations, which may influence their effects.

#### 3.3.2. Cancer

**Figure 3:**
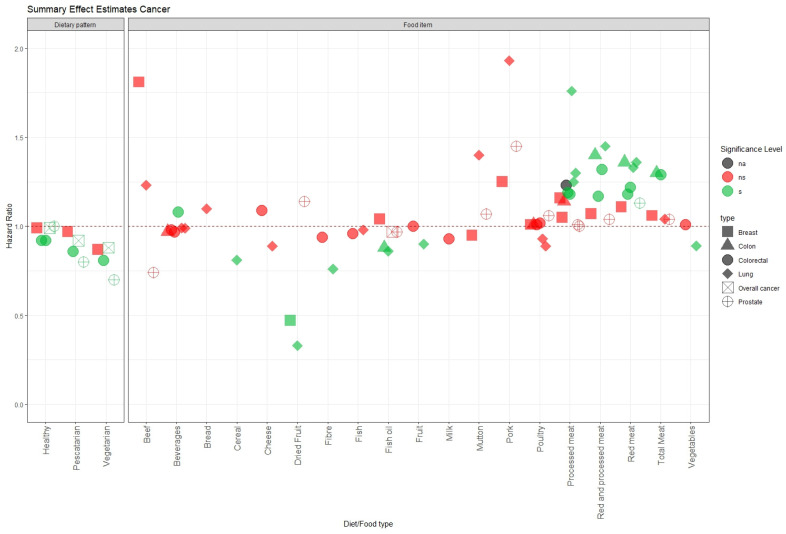
Summary of hazard ratios for the association between food/diet and cancer risk. Each point represents the hazard ratio for a specific food item or dietary pattern and a type of cancer. The shape shows what kind of cancer outcome was studied (square for breast cancer; triangle for colon cancer; circle for colorectal cancer; diamond for lung cancer; square cross for overall cancer; circle plus for prostate cancer). Horizontal reference line (red dashed) set at 1 indicates no association. The significance level is indicated by the colour of the point (grey for na = *p*-value not reported; red for ns = *p*-value ≥ 0.05; green for s = *p* value < 0.05).

Several studies have found a conclusive positive association between red meat or processed meat and colorectal cancer risk [23,27,28] (Figure 3). Moreover, some diet types that limit or exclude meat consumption (such as fish eaters and vegetarians) showed a beneficial effect on lowering cancer risk [29]. Studies that followed a healthy diet (with adequate fruits, vegetables, fish, and whole grains, and limited processed and red meats and refined grains) also observed a lower colorectal cancer risk [29,30]. This result is similar to those reported by the European Prospective Investigation into Cancer and Nutrition (EPIC) [55]. However, the results obtained by Wu et al. [30] suggest that genetics may be more important than diet in preventing colorectal cancer, as they observed a lower hazard ratio (HR) of 0.44 for low genetic risk and favourable lifestyle, compared to 0.73 for high genetic risk and favourable lifestyle, and 0.92 for healthy diet alone.

Most of the studies found no significant evidence or a mild association of their food group with any types of cancer [21,24,25,26]. A possible explanation for this might be in part due to the questionable selection of a single food group/type instead of a dietary pattern to detect association. Focus on certain beverages such as coffee [26] might not be a good predictor as the quantity of nutrient intake is too small to give a beneficial impact. While Jin et al. observed that dried fruit intake gave a meaningful decreased risk for breast and lung cancer [24], when looking at the confidence interval, the wide CI suggests a less precise estimate. 

#### 3.3.3. Type 2 Diabetes Mellitus (T2DM)

**Figure 4:**
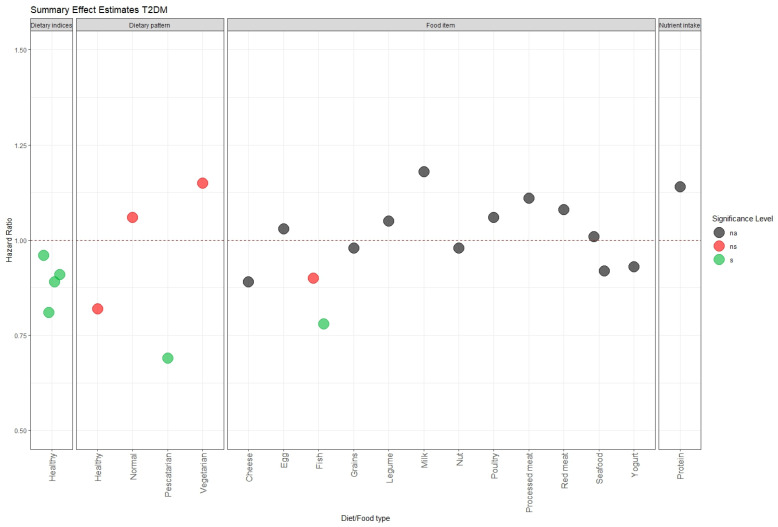
Summary of hazard ratio for the association between food/diet and type 2 diabetes mellitus risk. Each point corresponds to dietary pattern, food item, or nutrients. Horizontal reference line (red dashed line) set at 1 indicates no association. The significance level is shown by the colour of the point (grey for na = *p*-value not reported; red for ns = *p*-value ≥ 0.05; green for s = *p* value < 0.05).

Type 2 diabetes mellitus results from a combination of genetic and lifestyle factors, in which its development is thought to be greatly influenced by diet. The interest in finding links between diet and T2DM by combining UK Biobank data with other study cohorts has been high, in the hope of generating conclusive findings [32]. There is a clear message from the literature on T2DM and diet [34,35,36,37], which is in agreement with the wider research findings [56,57] that a healthy diet has potential for reducing the risk of diabetes. Briefly, a vegetarian diet showed no effect, but instead a balanced diet and high diet quality score were associated with lower diabetes risk [31,34,35,36,37] (Figure 4). A consideration of dietary pattern based on fat types showed no association with T2DM [33], while fish consumption was found to give different associations depending on the type of fish [38]. Unlike CVD, whose association with meat eating has been proven repeatedly in prospective cohort studies [58,59], the relation with T2DM is still vague [34,35,36,37,56,57].

#### 3.3.4. Range and Distribution of Healthy Diet, Red Meat, and Processed Meat

**Figure 5:**
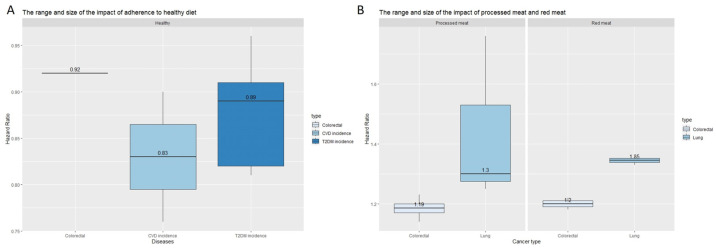
Summary effect estimates of diet on cancer, CVD, and T2DM risk: (**A**) Boxplot show the median HR of healthy diet on colorectal cancer, CVD incidence, and T2DM incidence. (**B**) Boxplot show the median HR of processed meat and red meat consumption on colorectal and lung cancer. The median for each effect estimate is indicated by horizontal lines and the value is shown inside the boxes.

Several studies have demonstrated that a healthy diet, red meat intake, and processed meat intake show consistent results on CVD, T2DM, and some cancer types (colorectal and lung). The median effect estimates were used to summarise the range and distribution of the observed effects. The median HRs of a healthy diet were 0.92 (interquartile range 0.92 to 0.92; n studies = 2), 0.83 (interquartile range 0.79 to 0.86; n studies = 3), and 0.89 (interquartile range 0.82 to 0.91; n studies = 5) for colorectal cancer, CVD incidence, and T2DM incidence, respectively (Figure 5a). The definitions of a healthy diet across these studies are varied, but they share common characteristics: consuming various food types, at least three servings/day of whole grains, fruits, and vegetables, and meat and processed meat less than twice a week. 

Processed meat and red meat increase the risk of lung cancer more than colorectal cancer based on the median HR (Figure 5b). Specifically, processed meat increases the risk of colorectal cancer and lung cancer by 18.5% (n studies = 4) and 30% (n studies = 4), respectively. Red meat consumption increases the risk of colorectal cancer and lung cancer by 20% (n studies = 2) and 34% (n studies = 2), respectively.

## 4. Discussion

The UK Biobank provides a variety of data on diet, enabling a study of its relationship with disease outcome, but this can only be achieved when conclusions on diet–disease relationships are drawn correctly. This is one of the benefits of meta-analyses. Thus, our analysis set out to summarise the findings for associations of diet with risk of three health outcomes in the UK Biobank population. Non-communicable diseases (NCDs) tend to result from genetic, physiological, environmental, and behavioural factors in combination. 

The Food Preference Questionnaire, which explores behavioural factors and has not been widely used in research, can in the future be analysed to reveal the diet–disease relationship in combination with entities like polygenic risk, or epigenomic characterisation scores derived for specific diseases. An example use of this questionnaire in the UK Biobank was given by May-Wilson et al. [60], who used hierarchical factor analysis (HFA) to find three clusters of foods (highly palatable, low caloric, and acquired food) and how different genetic predispositions contributed to these three food clusters. 

A comparison of the findings with those of other meta-analysis studies confirms that a high adherence to healthy diet guidelines provided by health authorities/organisations (e.g., WHO, AHA, ADA) appears to be protective for CVD, lung cancer, colorectal cancer, and T2DM [61,62,63,64,65]. The association between diet and cancer has been investigated by various systematic reviews and meta-analyses of observational and experimental studies, where a healthy diet score/pattern was mainly linked to a decreased risk of colorectal and lung cancer, but not all types of cancer [63,64,65]. This supports our finding that a cancer–diet association might only prevail in certain anatomical sites. These associations may reflect the biological mechanisms of these dietary factors on hormonal levels, inflammation, oxidative stress, insulin resistance, and microbiota composition, which are potential pathways for carcinogenesis [65]. The focus on healthy dietary patterns that incorporate a variety of foods, instead of a single food group/nutrient, give stronger associations for CVD and T2DM [61,62,66]. 

There is abundant room for further progress in incorporating multi-omics for investigating diet–disease associations. Walker et al. [67] found that certain proteins related to cell function and immunity correlated to diet. They also found that three healthy dietary patterns (Alternative Healthy Eating Index (AHEI), the Dietary Approaches to Stop Hypertension (DASH) diet, and a Mediterranean-style (MDS) diet) shared a common metabolic profile of 24 lipids. Another study found that AHEI was linked to specific fatty acids that lowered the risk of cardiovascular diseases [68], while MDS and DASH influenced glucose metabolism through circulating metabolites, which suggest prevention strategies for T2DM [69]. 

The studies we have seen to date have some limitations and challenges that need to be addressed in future research using UK Biobank data. First, the use of single dietary assessment methods for one time point only, such as FFQ or 24 h dietary recall, may introduce measurement errors and bias, especially when comparing results across studies. Second, the focus on single food groups or macronutrients may not capture the complexity and intricacy of dietary components. Moreover, the classification of dietary exposure into binary groups (i.e., ≥2/week vs. <2/week for meat intake) may oversimplify the dose–response relationship between diet and disease and ignore the potential threshold of intake. Combining several biomarker risk scores, including multi-omics biomarkers, or genetic risk scores with data on morbidities will provide a better prediction of the diet–disease relationship.

In conclusion, the effect of diet on different health outcomes was investigated in the current review. The results showed that a conventionally “healthy” dietary pattern had a significant impact but less than expected on the reduced risk of CVD, colorectal cancer, and T2DM. These findings suggest that diet may play an important role in preventing specific types of chronic diseases and shows that the UK Biobank cohort data agree with other epidemiological studies, validating their value for further research based on the multiple omics (epigenome, genome, proteome, metabolome) and questionnaire data available on UK Biobank volunteers.

## Figures and Tables

**Table 1:** Characteristics of studies included in the systematic review for CVD, cancer, T2DM, and other diseases in UK Biobank.

Author	Year	Number of Participants	FFQ	24 h Recall	Number of Repeated 24 h Recall	Dietary Focus
**Cardiovascular Diseases**						
McKenzie et al. [10]	2022	120,963	no	yes	2–5	Nutrient intake
Heianza et al. [11] ^a^	2020	156,148	no	yes	1–5	Dietary indices (healthy plant-based dietary patterns)
Brassard et al. [12] ^a^	2022	136,698	no	yes	1–5	Dietary indices (healthy eating food index recommended by CFG)
Petermann-Rocha et al. [13]	2021	422,791	yes	no	N/A	Dietary pattern
Chen, X. et al. [14]	2022	60,298	no	yes	1	The proportion of UPFs energy to total energy
Heianza et al. [15] ^a^	2021	121,799	no	yes	1–5	Dietary indices (healthy plant-based dietary patterns)
Ding et al. [16] ^a^	2022	346,627	yes	no	N/A	Dietary indices (diet quality index recommended by AHA)
Zhang, Y.B. et al. [17] ^a,b^	2021	399,537	yes	no	N/A	Diet indices (healthy eating index)
Livingstone et al. [18] ^a^	2021	77,004	no	yes	2–4	Dietary indices (healthy diet index recommended by WHO)
Feng et al. [19]	2022	399,586	yes	no	N/A	Vegetable intake
Ho et al. [20]	2020	195,658	no	yes	1–5	Nutrient intake
Cancer						
Wu, K. et al. [21] ^a^	2022	461,981	yes	no	N/A	Meat and processed meat intake
Wei et al. [22] ^a^	2021	416,588	yes	yes	1–5	Food group intake
Knuppel et al. [23] ^a^	2020	474,996	yes	yes	3–5	Total meat intake
Jin et al. [24] ^b^	2022	421,764	yes	no	N/A	Dried fruit intake
Liu, Z. et al. [25]	2022	470,804	yes	yes	1	Fish oil intake
Tran et al. [26]	2019	471,779	yes	no	N/A	Coffee intake
Bradbury et al. [27] ^a^	2020	475,581	yes	yes	1–5	Food group intake
Feng et al. [28] ^a^	2021	415,524	yes	no	N/A	Processed meat intake
Watling et al. [29] ^a^	2022	472,377	yes	no	N/A	Dietary pattern
Wu, E. et al. [30] ^a,b^	2022	390,365	yes	no	N/A	Dietary indices (diet quality index recommended by ACS)
**Type 2 Diabetes Mellitus**						
Boonpor et al. [31]	2022	203,790	yes	no	N/A	Dietary pattern
Li et al. [32] ^b^	2022	34,616	no	yes	4–5	Dietary protein group intake
Brayner et al. [33]	2021	16,523	no	yes	2–5	Dietary pattern
Andre et al. [34] ^a^	2020	21,585	no	yes	1–5	Dietary indices (adherence to a Mediterranean-style diet)
Song et al. [35] ^a^	2021	430,971	yes	no	N/A	Dietary indices (healthy diet index recommended by ADA)
Xu, C. et al. [36] ^a^	2022	59,849	no	yes	1	Dietary indices (EAT-LDP score)
Zhuang et al. [37] ^a^	2021	357,419	yes	no	N/A	Dietary indices (predefined diet quality score)
Chen, G.C. et al. [38]	2021	392,287	yes	yes	1–5	Fish intake
Gout						
Hutton et al. [39]	2018	130,966	yes	no	N/A	Coffee intake
Zhang, Y. et al. [40]	2022	416,481	yes	no	N/A	Dietary quality
**Inflammatory bowel disease**						
Chen, H. et al. [41]	2022	5763	yes	no	N/A	Meat intake
Huang et al. [42]	2022	447,890	yes	no	N/A	Fish oil intake
Fu et al. [43]	2022	121,490	no	yes	1–5	Sugar-sweetened beverage intake
**Rheumatoid arthritis**						
Mazzucca et al. [44]	2022	479,494	yes	no	N/A	Food and beverage group intake
Chen, W. et al. [45]	2022	335,576	no	yes	2	Beef intake

^a^ included in summary statistics; ^b^ studies in combination with other cohorts; ACS = American Cancer Society; ADA = American Diabetes Association; AHA = American Heart Association; CFG = Canada’s Food Guide; EAT-LDP = EAT-Lancet Dietary Pattern; FFQ= Food Frequency Questionnaire; UPFs = Ultra-Processed Foods; WHO = World Health Organization.

## Data Availability

No new data were created or analysed in this study. Data sharing is not applicable to this article.
